# High Thermoelectric Performance of Flexible and Free-Standing Composite Films Enabled by 3D Inorganic Ag_2_Se Conductive Networks Filled with Organic PVDF

**DOI:** 10.3390/polym17070972

**Published:** 2025-04-03

**Authors:** Zishuo Xu, Yuejuan Hu, Yuchen Hu, Xianfeng Xiao, Qin Yao

**Affiliations:** 1 State Key Laboratory of High-Performance Ceramics and Superfine Microstructure, Shanghai Institute of Ceramics, Chinese Academy of Sciences, Shanghai 200050, China; xuzishuo22@mails.ucas.ac.cn (Z.X.); huyuejuan23@mails.ucas.ac.cn (Y.H.); huych2022@shanghaitech.edu.cn (Y.H.); xiaoxf@shanghaitech.edu.cn (X.X.); 2 School of Physical Science and Technology, ShanghaiTech University, Shanghai 201210, China

**Keywords:** thermoelectric, silver selenide, polyvinylidene fluoride, free-standing, flexible

## Abstract

Herein, a flexible and free-standing (substrate-free) PVDF/Ag_2_Se (Polyvinylidene fluoride) composite film was successfully fabricated through a combination of drop-casting and heat treatment. It was observed that when the drop-casted PVDF/Ag_2_Se composite film was heated above the melting point of PVDF, the small and separated Ag_2_Se crystalline grains in the composite film grow and interconnect to form a three-dimensional (3D) conductive network to increase the carrier mobility, while the molten PVDF effectively fills the network voids to enhance the flexibility and mechanical strength. As a result, both the electrical conductivity and Seebeck coefficient of the composite films were significantly enhanced after heat treatment. The power factor of the PVDF/Ag_2_Se composite with a mass ratio of 1:4 at room temperature reached 488.8 μW m^−1^ K^−2^, among the best level of Ag_2_Se- or PVDF-based flexible and free-standing composite films. Bending tests demonstrated the superior flexibility of the hybrid film, with the electrical conductivity decreasing by only 10% after 1000 bending cycles. Additionally, a five-leg thermoelectric device achieved an impressive output power density of 1.75 W m^−2^ at a temperature difference (∆*T*) of 30 K. This study proposes an innovative strategy to enhance the thermoelectric performance and free-standing capability of organic-inorganic composite films, while achieving a competitive power factor and advancing the practical application of flexible thermoelectric devices.

## 1. Introduction

Thermoelectric (TE) materials, which can directly convert temperature gradients into electrical energy and vice versa, play a crucial role in renewable energy conversion [1]. Recently, the growing demand for applications such as wearable devices, microelectronics and Internet of Things has significantly driven the advancement of flexible thermoelectric materials, which are capable of powering electronics by harvesting heat from the human body or the environment, while also being adaptable to nonplanar surfaces [2,3,4]. The performance of thermoelectric materials is typically determined by the dimensionless figure of merit *ZT* (*ZT* = *σS*^2^/*κT*) or power factor (*PF* = *S*^2^*σ*), where *S*, *σ*, *κ* and *T* represent the Seebeck coefficient, electrical conductivity, thermal conductivity and absolute temperature, respectively [5].

Inorganic thermoelectric materials typically exhibit a high thermoelectric performance, but they are often brittle and rigid, which limits their application in flexible electronic devices [6,7]. On the other hand, organic thermoelectric materials possess excellent flexibility and processability, such as conducting polymers and their hybrids [8,9,10,11]. An organic–inorganic hybrid can combine the advantages of both and has been widely reported and studied as a vital approach to fabricate high-performance flexible thermoelectric materials [12,13,14].

TE materials can be categorized into p-type and n-type based on their charge carrier characteristics. The p-type flexible TE materials, such as nanocarbon materials, conducting polymers and conducting polymer-based composites, have been extensively studied and reported [15]. However, up to now, the development of n-type flexible materials has lagged behind their p-type counterparts [16]. For the completion of a high efficiency flexible TE module, high performance n-type materials are very desirable. Regarding inorganic filler, Ag_2_Se is a narrow bandgap N-type semiconductor material with a relatively higher power factor at room temperature and its TE performance has attracted widespread attentions [17,18]. Researchers have developed various methods, e.g., magnetron sputtering [19,20], vacuum-assisted filtration [21,22,23], screen printing [24,25], to prepare Ag_2_Se-based films by bonding Ag_2_Se on flexible substrates. Although most of these films exhibit promising TE performance, the weak bonding and the flexibility incompatibility between flexible substrates and Ag_2_Se materials challenge the heat-to-electricity conversion. In applications, the presence of the substrate commonly increases thermal loss, restricts electrical conductivity, introduces interfacial thermal resistance, and causes film cracking or delamination due to mechanical property mismatch. Therefore, it is essential to fabricate flexible and free-standing TE films based on Ag_2_Se and organic materials.

Polyvinylidene fluoride (PVDF) could be selected as a matrix for flexible TE materials due to its relatively low price and ease of processing [26]. So far, a variety of flexible PVDF-based free-standing composite films have been reported [27,28,29,30]. Dabin et al. prepared Ag_2_Se/PVDF composite film via a solution mixing method, demonstrating excellent thermoelectric performance (180.6 μW^−1^ K^−2^) and flexibility with 70 wt.% Ag_2_Se NWs [31]. Zhou et al. adopted a biomimetic approach to facilitate a high filler content (up to 90.5 wt%) in flexible n-type PVDF/Ag_2_Se thermoelectric films, achieving a power factor of 189.2 μW m^−1^ K^−2^ at 300 K [32]. Qu et al. rendered GDY as a bridge to connect the Ta_4_SiTe_4_ whiskers for good carrier transport in Ta_4_SiTe_4_/PVDF/GDY composite films, showing a maximum ZT value of 0.2 at 300 K [33]. Dun et al. added PVDF to grantee the robust and flexibility and create a high trap-state by introducing the energy barrier at the organic/inorganic interface in Cu-doped Bi_2_Se_3_/PVDF composite films, reaching a power factor of 103 μW m^−1^ K^−2^ at 290 K [29].

Herein, a facile strategy is proposed to fabricate flexible n-type free-standing Ag_2_Se/PVDF composite films through solution mixing Ag_2_Se nanowires and PVDF, followed by drop casting and then mechanically peeling the film from the glass substrate. It was found that the heat treatment process can significantly improve the thermoelectric performance of composite films. After heat treatment slightly above the melting point of PVDF, free-standing and flexible PVDF/Ag_2_Se composite films were successfully obtained. When the mass ratio of PVDF/Ag_2_Se reaches 1:4, an impressive power factor value of 488.8 μW m^−1^ K^−2^ is reached. The effects of Ag_2_Se content and heat temperature on the thermoelectric properties of composite film are investigated.

## 2. Experimental Section

### 2.1. Materials

Selenium oxide (SeO_2_ (IV), 99.4%), L-ascorbic acid (C_6_H_8_O_6_, 99+%) and silver nitrate (AgNO_3_ (I), 99.9+%) were purchased from Thermo Scientific (Shanghai, China). Poly (vinylidene fluoride) (PVDF, average Mw = ~534,000) and β-cyclodextrin (C_42_H_70_O_35_, 98%) were purchased from InnoCheme (Beijing, China). Ethylene glycol (C_2_H_6_O_2_, 99.5%), N,N-dimethylformamide (C_3_H_7_NO, 99.5%) and ethanol (C_2_H_5_OH, 99.5%) were purchased from Sinopharm Chemical Reagent Co (Shanghai, China). None of the chemicals were further purified.

### 2.2. Synthesis of Ag_2_Se NWs

Se NWs were first synthesized following a reported method described elsewhere [23]. Briefly, 1.000 g of SeO_2_, 1.000 g β-cyclodextrin and 4.000 g of C_6_H_8_O_6_ were dissolved in 400 mL of deionized water under stirring for 4 h to obtain Se NWs. The obtained Se NWs are washed by deionized water and ethanol alternately and dried at 80 °C for 24 h. The synthesis of Ag_2_Se NWs was carried out according to a procedure reported elsewhere [23]. Typically, the as-prepared Se NWs (0.100 g) and AgNO_3_ (0.430 g) were dispersed in 200 mL of ethylene glycol. Then, the aqueous solution of C_6_H_8_O_6_ is added with the molar ratio m (AgNO_3_): m (C_6_H_8_O_6_) = 1:3 and vigorous stirring for 4 h. The product is washed by deionized water and ethanol 3 times. Afterward, Ag_2_Se NWs were dried in a vacuum oven for the following experiments.

### 2.3. Fabrication of Ag_2_Se/PVDF Free-Standing Flexible TE Films

The as-prepared Ag_2_Se NWs and PVDF with different ratios were dissolved in dimethylformamide (DMF) by alternately performing bath sonication and vigorous stirring. The uniform suspension is drop-coated on a 9 × 18 mm^2^ glass plate and then dried at 80 °C in a hot bench for 24 h to obtain Ag_2_Se/PVDF composite films by removing the DMF. During the heat treatment process, the dried Ag_2_Se/PVDF composite films were heated at different temperature for half an hour. PVDF/Ag_2_Se free-standing flexible TE films were finally obtained by peeling off these thin films from the glass substrate. The mass ratio between PVDF and Ag_2_Se in the PVDF/Ag_2_Se films varies from 1:0.5, 1:1, 1:2, 1:3, 1:4 and 1:5. The corresponding samples before heat treatment were named 1-0.5-BH, 1-1-BH, 1-2-BH, 1-3-BH, 1-4-BH and 1-5-BH, respectively. The PVDF/Ag_2_Se films with the mass ratio of 1:4 was heated under 115 °C, 145 °C, 175 °C, 205 °C and 235 °C, respectively, named 1-4-AH115, 1-4-AH145, 1-4-AH175, 1-4-AH205 and 1-4-AH235. The thickness of these PVDF/Ag_2_Se films was kept at around 10 μm.

### 2.4. Materials Characterization

X-ray diffraction (XRD) data were collected using a Bruker D8 Advance (Berlin, Germany) with a Cu Kα source by a scanning speed of 1° min^−1^. The morphologies were measured using a high-resolution field emission scanning electron microscope (FESEM, Verios G4, FEIC, Hillsboro, OR, USA) equipped with energy-dispersive spectroscopy (EDS, Oxford Horiba 250, Portland, OR, USA) and field emission transmission electron microscope (TEM, JEM-2100F, JEOL, Tokyo, Japan). The thickness is measured by a step meter (Dektak-XT, Bruker, Berlin, Germany). Thermal diffusion coefficients are measured by a laser pulse analyzer (LFA-467, Netzsch, Bavaria, Germany) through in-plane mode. Specific heat capacity (*C_p_*) and material thermal properties (melting point) are measured by a Differential Scanning Calorimeter DSC8000 (DSC8000, PerkinElmer, Waltham, MA, USA). The densities (*D*) were measured by the drainage method. The thermal conductivity was calculated by the following equation *κ* = *ρ*·*C_p_*·*D*.

### 2.5. Thermoelectric Property and Flexibility Testing

The electrical conductivity and Seebeck coefficient are simultaneously measured by the Seebeck coefficient/resistance measurement system (NETZSCH, SBA458, Bavaria, Germany). The flexibility of films is evaluated by bending the films onto the surface of cylinders with a specific bending radius of 5 mm. The electrical resistance (*R*) at room temperature before and after bending was measured by a portable Ohmmeter, and the electrical conductivity was then calculated through *L*/(*R* × *A*), where *A* is the cross-section area and *L* is the length of the films.

### 2.6. Performance Evaluation of the Flexible Thermoelectric Device (FTD)

The output performance of FTD was measured through a homemade instrument. By heating one side of the FTD, a stable temperature gradient is established between the two sides of the FTD. By adjusting the load resistance, the output voltage (*V*) and output current (*I*) were measured. The details of fabrication and performance measurement of the FTD are illustrated in Note S1.

## 3. Results and Discussion

**Figure 1:**
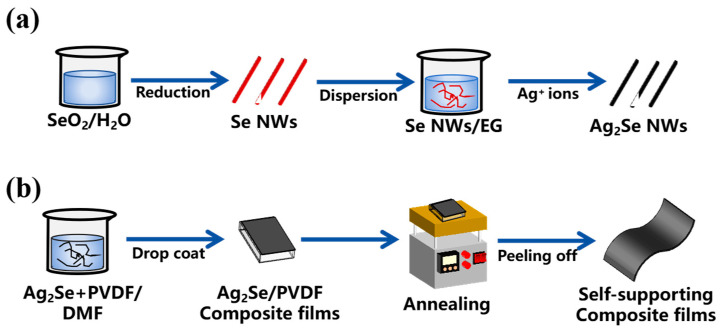
Schematic illustration for the preparation of (**a**) Ag_2_Se NWs and (**b**) free-standing and flexible PVDF/Ag_2_Se composite TE films.

**Figure 2:**
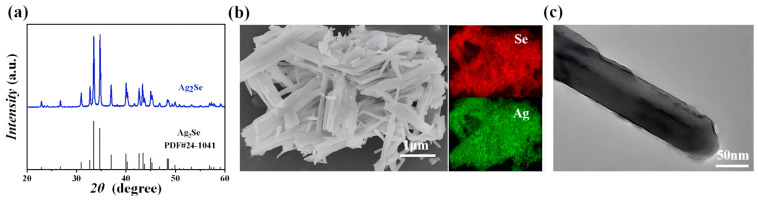
(**a**) XRD pattern, (**b**) SEM image with EDS mappings of Se and Ag atoms and (**c**) TEM image of Ag_2_Se NWs.

The summarized experimental procedure is shown in Figure 1 (detailed experimental procedures are provided in Section 2). As shown in Figure 2a, all the peaks correspond to the Ag_2_Se orthorhombic structure (JCPDS no. 24-1041). The SEM and TEM images (Figure 2b or Figure 2c) exhibited most of the Ag_2_Se nanowires range in length from 500 nm to 2 µm with diameter around 70 nm. The EDS mappings confirm that the elemental Se and Ag in the Ag_2_Se nanowires are uniformly distributed without obvious element enrichment.

**Figure 3:**
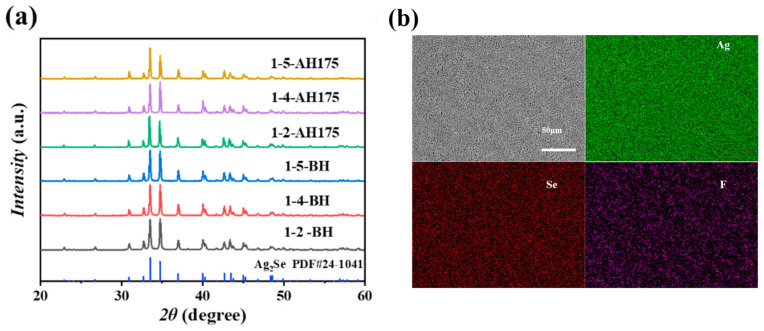
(**a**) XRD patterns of PVDF/Ag_2_Se composite films with mass ratio 1:2, 1:4 and 1:5 before and after heat treatment. (**b**) EDS mappings of PVDF/Ag_2_Se composite film with a mass ratio 1:4 after heat treatment at 175 °C.

Figure 3a shows the XRD patterns of pure Ag_2_Se film and Ag_2_Se/PVDF composite films with different mass ratios. It can be seen, before and after heat treatment, that all characteristic diffraction peaks of composite film correspond to the orthorhombic structure of Ag_2_Se (JCPDS no. 24-1041). This indicates that neither the heat treatment process nor the PVDF compositing affected the chemical composition or crystal structure of the Ag_2_Se nanowires, with no detectable impurity phases formed in the composite film [34]. The EDS mapping of elemental Se, Ag and F further confirmed that Ag_2_Se nanowires uniformly distributed in the PVDF (Figure 3b).

**Figure 4:**
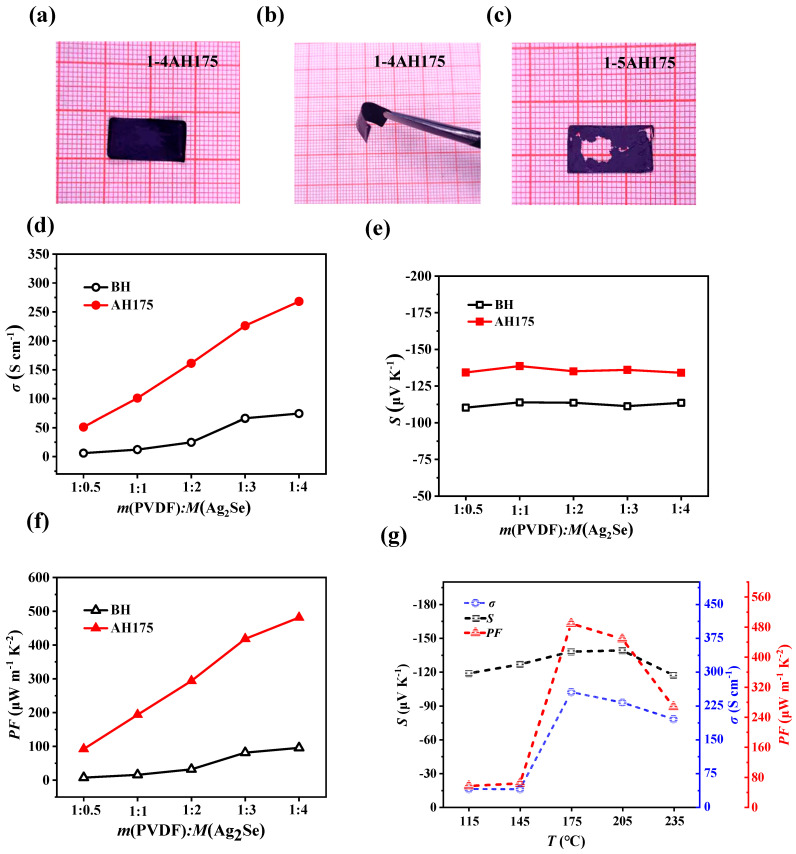
(**a**–**c**) Digital photographs of PVDF/Ag_2_Se composite film with mass ration 1:4 and 1:5 peeled from the glass substrate. (**d**) Electrical conductivity, (**e**) Seebeck coefficient and (**f**) power factor of PVDF/Ag_2_Se composite films with different mass ratio before (BH) and after heat treatment at 175 °C (AH175). (**g**) Electrical conductivity, Seebeck coefficient and power factor of PVDF/Ag_2_Se composite films with a mass ratio of 1:4 after heat treatment at different temperatures.

When we attempted to peel the Ag_2_Se/PVDF composite films from the glass substrate, it was found that when the mass ratio of m (PVDF)_:_M (Ag_2_Se) ≤ 1:4, the films possessed enough high mechanical strength and flexibility, allowing them to be successfully peeled off to obtain free-standing films (Figure 4a,b). However, when the ratio of m (PVDF)_:_ M (Ag_2_Se) reaches 1:5, the mechanical strength and flexibility of the film significantly decreased due to the low content of PVDF, leading to fragmentation during the peeling process (Figure 4c).

**Figure 5:**
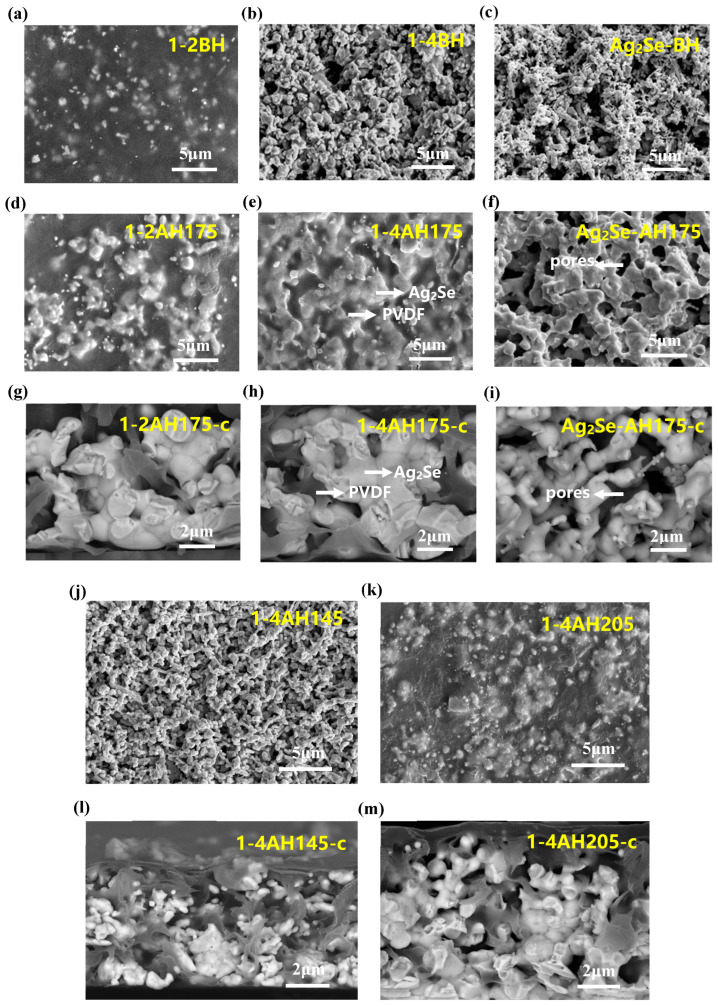
The Surface SEM images of PVDF/Ag_2_Se composite films with mass ratios of 1:2, 1:4 and pure Ag_2_Se film before (**a**–**c**) and after heat treatment at 175 °C (**d**–**f**); the cross-section SEM images of PVDF/Ag_2_Se composite films with mass ratios of 1:2, 1:4 and pure Ag_2_Se film after heat treatment at 175 °C (**g**–**i**); the Surface (**j**,**k**) and cross-section (**l**,**m**) SEM images of PVDF/Ag_2_Se composite films with mass ratios of 1:4 after heat treatment at 145 °C and 205 °C, respectively. (The bright white areas represent Ag_2_Se, the gray-black areas are PVDF, and the dark black areas are pores, as indicated by the arrows).

Subsequently, we measured the thermoelectric properties of the composite films with m (PVDF)_:_M (Ag_2_Se) ≤ 1:4, as shown in Figure 4d–f. The electrical conductivity (*σ*) increases with the increase of Ag_2_Se content, while the Seebeck coefficient (*S*) remains stable, exhibiting minimal variation with the increase in Ag_2_Se content. For the PVDF/Ag_2_Se composite films, the PVDF is an insulating polymer, thus charge carriers can only transport through the conductive network formed by Ag_2_Se. At high Ag_2_Se loading (the mass ratio ≥ 1:0.5, i.e., Ag_2_Se wt% > 33.3%), the formation of continuous conductive network (as shown in Figure 5g,h) allows the charge transport to be dominated by Ag_2_Se’s intrinsic band characteristics (rather than interfacial effects), causing the *S* to approach the bulk value of Ag_2_Se independently of PVDF content. Thus, the *S* remains almost constant with the increase of Ag_2_Se content [35]. However, after heat treatment at 175 °C, both the *σ* and *S* of composite films were significantly improved. For example, the *σ* of 1-4 samples increased from 74.1 S cm^−1^ to 255.2 S cm^−1^ after heat treatment, representing an increase of more than three times. Before heat treatment, the *S* of these composite films was ~−113.5 μV K^−1^, and after heat treatment, it increased to ~−138.4 μV K^−1^. Consequently, the maximum power factor of PVDF/Ag_2_Se composite film reached 488.8 μW m^−1^ K^2^ for the sample with mass ratio 1:4 after heat treatment at 175 °C (1-4-AH175), which is among the best level of Ag_2_Se–based or PVDF-based free-standing composite flexible films (Table 1). The in-plane thermal conductivity of 1-4-AH175 is 0.36 W m^−1^ K^−1^ at room temperature, and the estimated ZT value reached 0.41, which is a remarkably high figure of merit for organic/inorganic flexible thermoelectric materials.

As shown in Figure 5, before heat treatment, the particle size of Ag_2_Se grains was small, about 200~500 nm; after heat treatment, the grains remarkably grew, reaching sizes of 2 to 3 μm (Figure 5a,b,d,e). During the heat treatment process, Ag_2_Se grains undergo recrystallization and growth, resulting in a significant increase in grain size [43,44]. With the increase of Ag_2_Se content, the larger grains interconnect to form a conductive network (Figure 5e), which leads to great increase in carrier mobility [45], and therefore both the *σ* and the *S* were improved remarkably after heat treatment [43,44] (Figure 4d,e). As shown in Figure S2, with the increase of Ag_2_Se content, the carrier mobility remained stable, but after heat treatment at 175 °C, the carrier mobility remarkably increased, from ~60 cm^2^V^−1^s^−1^ to ~560 cm^2^V^−1^s^−1^. On the other hand, for heat-treated pure Ag_2_Se film, there are many pores in the Ag_2_Se conductive network (Figure 5f,i), which results in the material exhibiting brittle and fragile characteristics. These pores not only reduce the mechanical strength of the material but may also affect the transport path of charge carriers, thereby adversely affecting conductivity and thermoelectric properties. For heat-treated PVDF/Ag_2_Se composite film (Figure 5g,h), the PVDF intersperses and fills within the conductive network of Ag_2_Se, providing the composite film with excellent mechanical support and flexibility [28]. Additionally, the clear distribution of Ag_2_Se, PVDF and pores within the PVDF/Ag_2_Se composite film (1-4AH175-c) and pure Ag_2_Se film (Ag_2_Se-AH175-c) was provided by the EDS mappings (Figure S3). This structure not only enhances the durability and adaptability of the film but also maintains the integrity of the conductive pathways, ensuring efficient electrical conductivity throughout the material. The inherent properties of PVDF, such as its mechanical strength and flexibility, complement the brittle nature of Ag_2_Se [28], resulting in a composite that is both mechanically robust and functionally superior for thermoelectric applications.

We further investigated the effect of heat treatment temperature on the thermoelectric properties of the PVDF/Ag_2_Se composite films with a mass ratio of 1:4. As shown in Figure 4g, when the heat treatment temperature is at 115 °C and 145 °C, the *σ* does not show significant changes after heat treatment. When the heat treatment temperature reaches the 175 °C, the *σ* of the composite film increases sharply. However, as the heat treatment temperature continues to rise, the *σ* subsequently decreases. The *S* also initially increases, reaching a peak value around 175 °C, and then decreases as the heat treatment temperature rises. Finally, the thermoelectric power factor attains its maximum value at a heat treatment temperature of 175 °C.

The surface and cross-sectional morphologies of the films subjected to different heat treatment temperatures were characterized using SEM (as shown in Figure 5e,h,j–m). From the DSC curve of PVDF (Figure S1), it can be observed that an endothermic peak appears near 166 °C, indicating that its melting point (*T_m_*) is around 166 °C. When the *T* < *T_m_* (e.g., 145 °C), the size of Ag_2_Se grains in the composite film shows no significant change after heat treatment, presenting a loose and porous structure like its pre-treatment morphology (Figure 5j,l). This indicates that low-temperature heat treatment is insufficient to grain growth of Ag_2_Se. As a result, the *σ* and *S* of the composite film remain unchanged compared to those before heat treatment. When *T* > *T_m_* (e.g., 175 °C), the Ag_2_Se grains grow significantly and become interconnected, while molten PVDF fills the pores in the film, leading to a denser structure (Figure 5e,h). This suggests that the material undergoes significant grain growth, and the enhanced fluidity of PVDF effectively fills the voids, forming a dense structure conducive to charging carrier transport. Consequently, the *σ* and *S* of the composite film increase. When the *T* ≫ *T_m_* (e.g., 205 °C), the size of the Ag_2_Se grains decrease, and the number of pores in the film increases (Figure 5k,m), which may be due to the partial decomposition or remelting of Ag_2_Se grains caused by excessively high temperatures. Notably, the element Se will melt and decompose from Ag_2_Se above 221 °C (Figure S1). This leads to a more porous and less dense film structure. Accordingly, the *σ* and *S* of the composite films decrease. The above research results demonstrate that the heat treatment temperature significantly affects the structure and electrical transport properties of PVDF/Ag_2_Se composite films.

**Figure 6:**
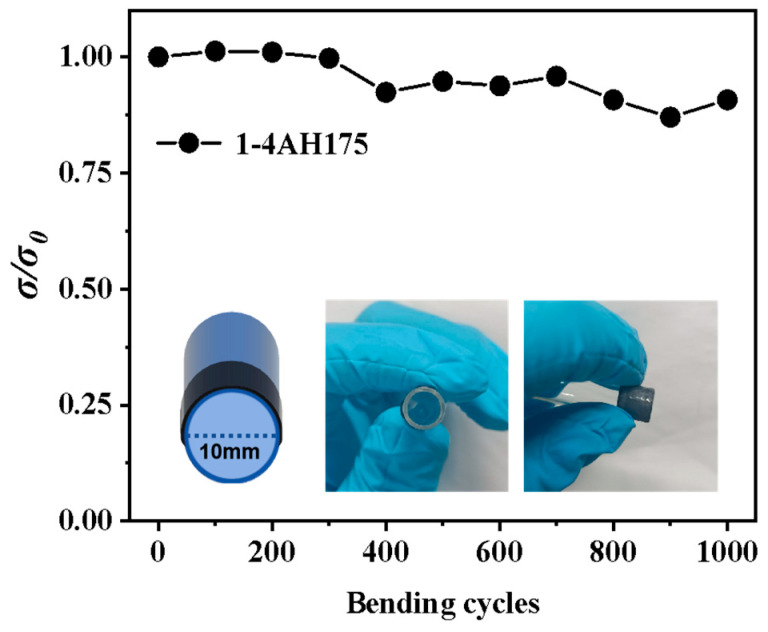
The relative electrical conductivity (σ/σ_0_) of the 1-4AH175 PVDF/Ag_2_Se composite film before and after bending as a function of bending cycles with a specific bending radius 5 mm.

In order to test the flexibility of the PVDF/Ag_2_Se film, the 1-4AH175 sample is bent to different cycles with a specific bending radius of 5 mm and the relative electrical conductivity (*σ*/*σ*_0_) of the film before and after bending is measured. As seen in Figure 6, the film maintains approximately 90% of its original conductivity after 1000 bending cycles, demonstrating excellent flexibility.

**Figure 7:**
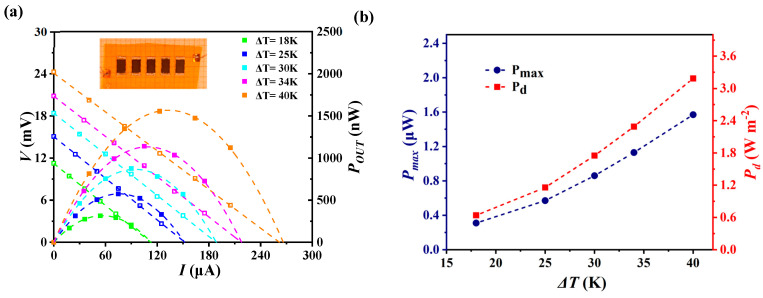
Performance of the prepared TE device with the 1-4AH175 PVDF/Ag_2_Se composite film. (**a**) Output voltage and power versus current at different ∆*T*. (**b**) Maximum output power and power density value at different ∆*T*.

A single-leg thermoelectric module with 9 × 18 mm^2^ was fabricated using 1-4AH175 composite film. Figure 7a presents output voltage and power versus current at different ∆*T*. The corresponding maximum output power density (*P_d_*) at different Δ*T* are shown in Figure 7b. When the Δ*T* is 18, 24, 30, 35 and 40 K, the corresponding measured maximum *P_d_* is 0.64, 1.16, 1.75, 2.29 and 3.19 W m^−2^, respectively. This composite film exhibits an impressive *P_d_* compared with the previous reports (Table 1), indicating the great application potential of our device.

## 4. Conclusions

In summary, free-standing and flexible n-type PVDF/Ag_2_Se composite films have been successfully fabricated through a simple process involving drop casting, heat treatment and peeling the film from the substrate. The electrical conductivity increases with the rising mass ratio of PVDF to Ag_2_Se. The heat treatment process significantly enhances the electrical conductivity and Seebeck coefficient of the composite films while preserving their flexibility. SEM characterization results reveal that when the PVDF/Ag_2_Se composite film is heated slightly above the melting point of PVDF (175 °C), the Ag_2_Se grains grow and interconnect to form a three-dimensional conductive network. Simultaneously, the molten and flowing PVDF effectively fills the network voids, creating a dense structure that not only facilitates carrier transport but also enhances the film’s flexibility and mechanical strength. As a result, the composite film exhibits a significant improvement in both electrical conductivity and the Seebeck coefficient while maintaining excellent flexibility and free-standing ability. When the PVDF/Ag_2_Se composite film with a mass ratio of 1:4 is heat-treated at 175 °C (denoted as 1-4AH175), its thermoelectric power factor reaches a maximum of 488.8 μWm^−1^K^−2^, ranking among the highest values reported for Ag_2_Se-based flexible and free-standing composite films. Additionally, after 1000 bending cycles at a bending radius of 5 mm, the (*σ*/*σ*_0_) of the 1-4AH175 composite film decreases by only 10%, demonstrating competitive performance compared to other flexible thermoelectric films. Furthermore, a flexible device based on the 1-4AH175 composite film has been fabricated, achieving an impressive output power density of 1.75 Wm^−2^ at a temperature difference (∆*T*) of 30 K. This work presents a novel strategy for enhancing the thermoelectric performance and free-standing ability of organic/inorganic flexible composite films, offering a competitive power factor and advancing the practical application of organic/inorganic flexible thermoelectric devices at room temperature.

## Figures and Tables

**Table 1:** The typical TE performances of Ag_2_Se-based or PVDF-based free-standing and flexible composite films at room temperature (300 K).

Films	S (μV K^−1^)	σ (S cm^−1^)	PF (μW m^−1^ K^−2^)	P_d_ (W m^−2^)	Refs.
PVP/Ag_2_Se	−58.5	~	4.3 (390 K)	0.08 (40 K)	[36]
PPy/Ag_2_Se	~	590.9	282.8	~	[37]
PVDF/PANI-coated Ag_2_Se	~	~	196.6	2.33 (30 K)	[27]
PVDF/Ag_2_Se	−95.9	205.52	189.02	0.0098 (30 K)	[32]
PVDF/Ag_2_Se	~	~	180.6 (400 K)	~	[31]
Ni/PVDF	−20.6	~	200	~	[30]
Ag_2_Te/PVDF	−60	86	30.9	~	[38]
Cu-doped Bi_2_Se_3_/PVDF	−84	146	103	~	[29]
TaSiTe_4_/GDY/PVDF	~	113	489	6 (35 K)	[33]
FLG/PVDF	18.3	20.05	0.52	~	[39]
Cu_1.75_Te NWs/PVDF	9.6	2490	23	~	[40]
BC/Ag_2_Se	−171	132	386	0.0047 (25 K)	[41]
Bi_0.5_Sb_1.5_Te_3_ (BST)/PVDF Bi_0.5_Te_2.7_Se_0.3_ (BTS)/PVDF	112 −96	0.74 0.50	0.94 0.47	0.00025 (20 K)	[42]
This work	−138.4	255.2	488.8	1.75 (30 K) 3.19 (40 K)	

## Data Availability

The original contributions presented in this study are included in the article and Supplementary Materials. Further inquiries can be directed to the corresponding author.

## Supplementary Materials

The following supporting information can be downloaded at https://www.mdpi.com/article/10.3390/polym17070972/s1, Figure S1: The DSC curve of pure PVDF, Ag2Se, Se and 1-4BH PVDF/Ag_2_Se composite film; Figure S2: (a) Carrier concentration and carrier mobility of PVDF/Ag_2_Se composite films with a mass ratio of 1:2, 1:3, 1:4 before and after heat treatment at 175 °C; Figure S3: EDS mappings of (a) PVDF/Ag_2_Se composite film with a mass ratio 1:4 and (b) pure Ag_2_Se film after heat treatment at 175 °C. Note S1: Fabrication of Flexible Thermoelectric Device (FTD).
