# Supplementary material for: High Thermoelectric Performance of Flexible and Free-Standing Composite Films Enabled by 3D Inorganic Ag2Se Conductive Networks Filled with Organic PVDF
Source: Polymers (Basel). 2025 Apr 3;17(7):972. doi: 10.3390/polym17070972 (PMC11991616; doi:10.3390/polym17070972)
Supplement: Supplementary file 1 [file polymers-17-00972-s001.zip › polymers-3541371-supplementary.pdf]

Supporting Information

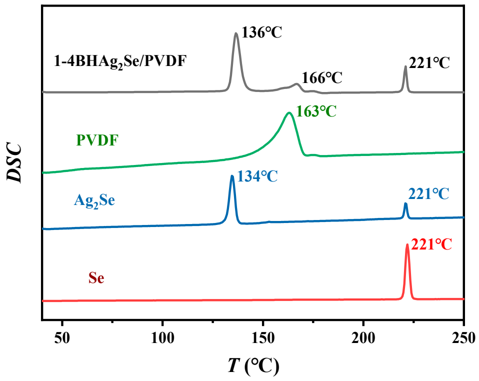

Figure S1. The DSC curve of pure PVDF, Ag_2Se, Se and 1-4BH PVDF/Ag_2Se composite film.

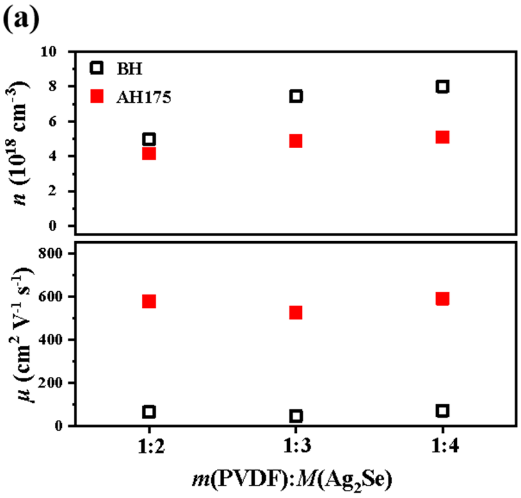

Figure S2. (a) Carrier concentration and carrier mobility of PVDF/Ag_2Se composite films with a mass ratio of 1:2, 1:3, 1:4 before and after heat treatment at 175°C.

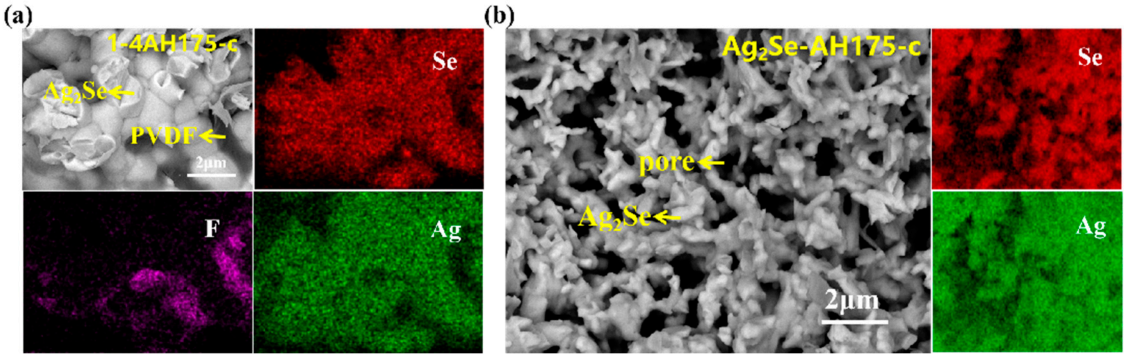

Figure S3. EDS mappings of (a) PVDF/Ag_2Se composite film with a mass ratio 1:4 and (b) pure Ag_2Se film after heat treatment at 175 °C.

Note S1.

Fabrication of Flexible Thermoelectric Device (FTD): The FTD was composed of five legs (width×length of 9×18mm^2) of Ag_2Se/PVDF composite films with a mass ratio of 1:4 after heat-treated at 175°C. The average thickness of the composite film was 0.011mm. Two ends of each leg were covered by Ag paste and connected by Pt wires in series. The array distance between legs was 6 mm.

Performance Evaluation of the FTD: The device was connected into an electrical circuit with a multimeter (Agilent 34970A) and a constant current source (Agilent B2901A). The output voltages under different applied currents were recorded by multimeter. The power output was obtained by multiplying the current and corresponding voltage. The ΔT was controlled by a homemade device. The hot sides were heated by heating plates and the cold side was maintained at 30°C. The temperature was calibrated using the thermocouple.

The output power (P) of the module is calculated by using the following expression:

$$P = UI = \frac{U^2 R_{ex}}{(R_{in}+R_{ex})^2} \quad (1)$$

where U is the output voltage, I is the output current, R_{in} is the internal resistance of the module, R_{ex} is the external resistance. When the R_{ex} value equals R_{in}, the maximum power (P_{max}) is obtained.

The maximum power density (P_d) can be calculated by using the following equation:

$$P_d = \frac{P_{max}}{Nwd} \quad (2)$$

where N, w, and d are the number, width and thickness of the TE leg.

The maximum normalized power density (P_D) can be calculated by using the following equation

$$P_D = \frac{P_d L}{\Delta T^2} \quad (3)$$

where L is the length of the TE leg.
